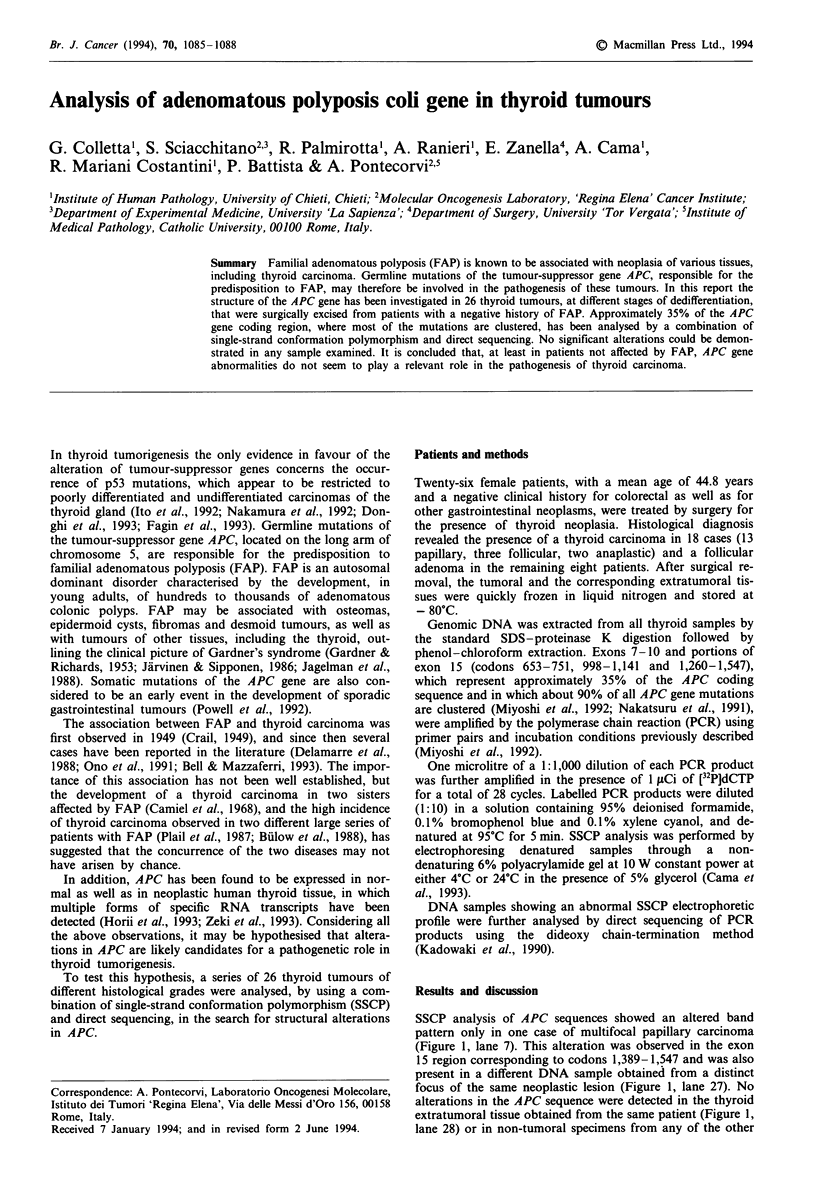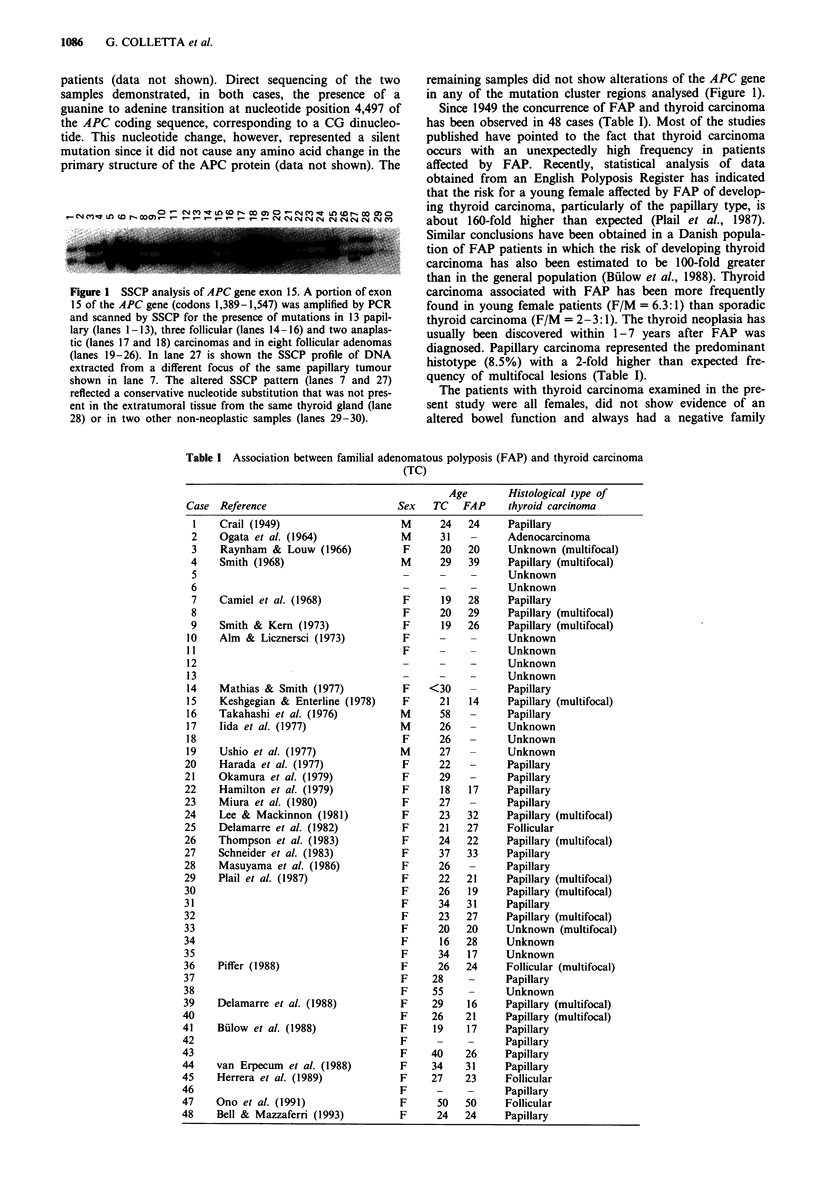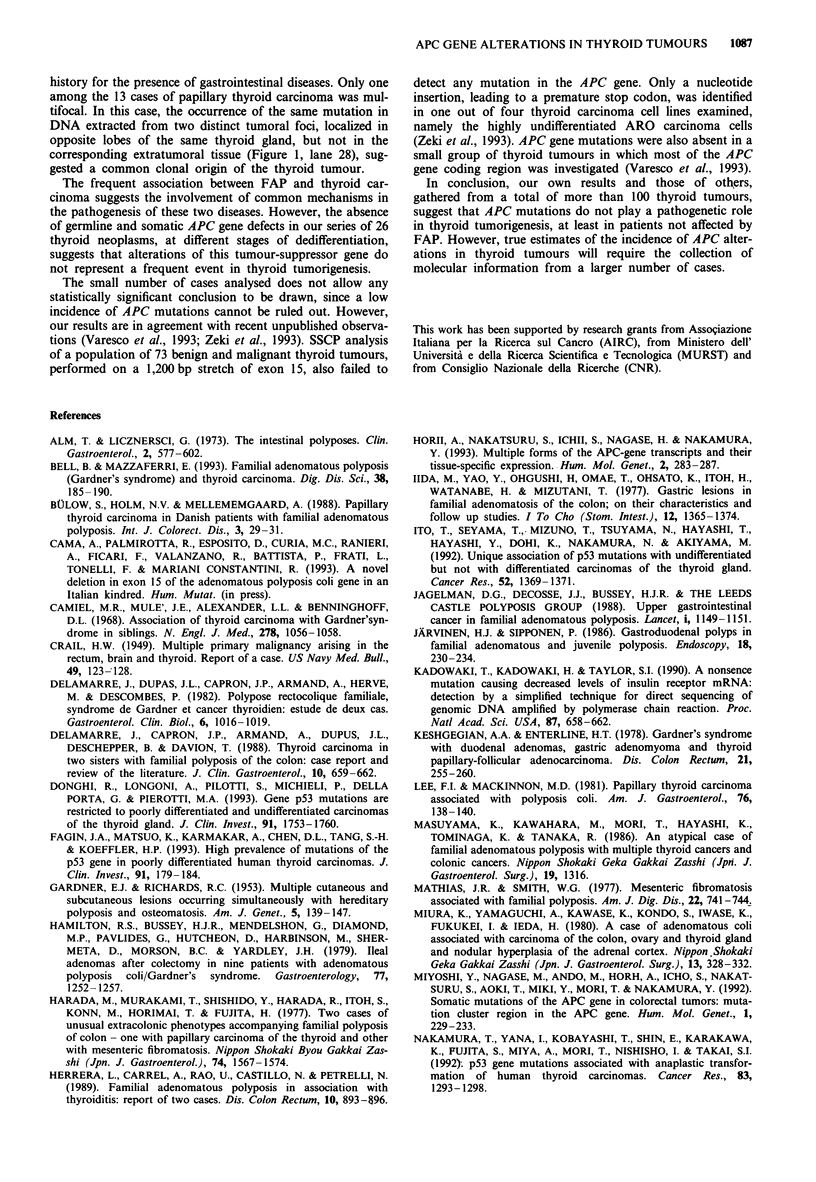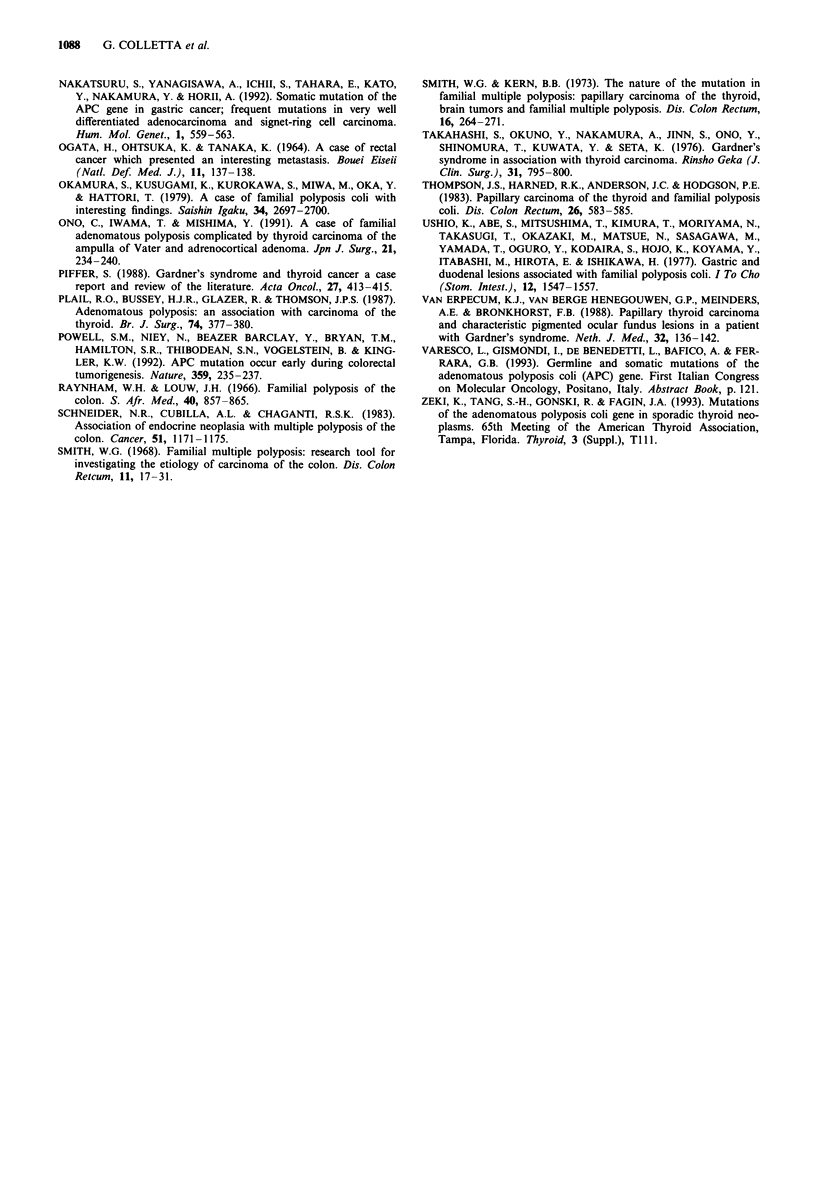# Analysis of adenomatous polyposis coli gene in thyroid tumours.

**DOI:** 10.1038/bjc.1994.452

**Published:** 1994-12

**Authors:** G. Colletta, S. Sciacchitano, R. Palmirotta, A. Ranieri, E. Zanella, A. Cama, R. Mariani Costantini, P. Battista, A. Pontecorvi

**Affiliations:** Institute of Human Pathology, University of Chieti, Italy.

## Abstract

**Images:**


Br. J. Cancer (1994), 70, 1085-1088 © Macmillan Press Ltd., 1994

### Analysis of adenomatous polyposis coli gene in thyroid tumours

G. Colletta[1], S. Sciacchitano[2,3], R. Palmirotta[1], A. Ranieri[1], E. Zanella[4], A. Cama[1], R. Mariani Costantini[1], P. Battista & A. Pontecorvi[2,5]

[1]*Institute of Human Pathology, University of Chieti, Chieti;* [2]*Molecular Oncogenesis Laboratory, 'Regina Elena' Cancer Institute;* [3]*Department of Experimental Medicine, University 'La Sapienza';* [4]*Department of Surgery, University 'Tor Vergata';* [5]*Institute of Medical Pathology, Catholic University, 00100 Rome, Italy.*

**Summary** Familial adenomatous polyposis (FAP) is known to be associated with neoplasia of various tissues, including thyroid carcinoma. Germline mutations of the tumour-suppressor gene *APC*, responsible for the predisposition to FAP, may therefore be involved in the pathogenesis of these tumours. In this report the structure of the *APC* gene has been investigated in 26 thyroid tumours, at different stages of dedifferentiation, that were surgically excised from patients with a negative history of FAP. Approximately 35% of the *APC* gene coding region, where most of the mutations are clustered, has been analysed by a combination of single-strand conformation polymorphism and direct sequencing. No significant alterations could be demonstrated in any sample examined. It is concluded that, at least in patients not affected by FAP, *APC* gene abnormalities do not seem to play a relevant role in the pathogenesis of thyroid carcinoma.

In thyroid tumorigenesis the only evidence in favour of the alteration of tumour-suppressor genes concerns the occurrence of p53 mutations, which appear to be restricted to poorly differentiated and undifferentiated carcinomas of the thyroid gland (Ito *et al.*, 1992; Nakamura *et al.*, 1992; Donghi *et al.*, 1993; Fagin *et al.*, 1993). Germline mutations of the tumour-suppressor gene *APC*, located on the long arm of chromosome 5, are responsible for the predisposition to familial adenomatous polyposis (FAP). FAP is an autosomal dominant disorder characterised by the development, in young adults, of hundreds to thousands of adenomatous colonic polyps. FAP may be associated with osteomas, epidermoid cysts, fibromas and desmoid tumours, as well as with tumours of other tissues, including the thyroid, outlining the clinical picture of Gardner's syndrome (Gardner & Richards, 1953; Järvinen & Sipponen, 1986; Jagelman *et al.*, 1988). Somatic mutations of the *APC* gene are also considered to be an early event in the development of sporadic gastrointestinal tumours (Powell *et al.*, 1992).

The association between FAP and thyroid carcinoma was first observed in 1949 (Crail, 1949), and since then several cases have been reported in the literature (Delamarre *et al.*, 1988; Ono *et al.*, 1991; Bell & Mazzaferri, 1993). The importance of this association has not been well established, but the development of a thyroid carcinoma in two sisters affected by FAP (Camiel *et al.*, 1968), and the high incidence of thyroid carcinoma observed in two different large series of patients with FAP (Plail *et al.*, 1987; Bülow *et al.*, 1988), has suggested that the concurrence of the two diseases may not have arisen by chance.

In addition, *APC* has been found to be expressed in normal as well as in neoplastic human thyroid tissue, in which multiple forms of specific RNA transcripts have been detected (Horii *et al.*, 1993; Zeki *et al.*, 1993). Considering all the above observations, it may be hypothesised that alterations in *APC* are likely candidates for a pathogenetic role in thyroid tumorigenesis.

To test this hypothesis, a series of 26 thyroid tumours of different histological grades were analysed, by using a combination of single-strand conformation polymorphism (SSCP) and direct sequencing, in the search for structural alterations in *APC*.

Correspondence: A. Pontecorvi, Laboratorio Oncogenesi Molecolare, Istituto dei Tumori 'Regina Elena', Via delle Messi d'Oro 156, 00158 Rome, Italy.
Received 7 January 1994; and in revised form 2 June 1994.

### Patients and methods

Twenty-six female patients, with a mean age of 44.8 years and a negative clinical history for colorectal as well as for other gastrointestinal neoplasms, were treated by surgery for the presence of thyroid neoplasia. Histological diagnosis revealed the presence of a thyroid carcinoma in 18 cases (13 papillary, three follicular, two anaplastic) and a follicular adenoma in the remaining eight patients. After surgical removal, the tumoral and the corresponding extratumoral tissues were quickly frozen in liquid nitrogen and stored at − 80°C.

Genomic DNA was extracted from all thyroid samples by the standard SDS-proteinase K digestion followed by phenol-chloroform extraction. Exons 7-10 and portions of exon 15 (codons 653-751, 998-1,141 and 1,260-1,547), which represent approximately 35% of the *APC* coding sequence and in which about 90% of all *APC* gene mutations are clustered (Miyoshi *et al.*, 1992; Nakatsuru *et al.*, 1991), were amplified by the polymerase chain reaction (PCR) using primer pairs and incubation conditions previously described (Miyoshi *et al.*, 1992).

One microlitre of a 1:1,000 dilution of each PCR product was further amplified in the presence of 1 μCi of [$^{32}$P]dCTP for a total of 28 cycles. Labelled PCR products were diluted (1:10) in a solution containing 95% deionised formamide, 0.1% bromophenol blue and 0.1% xylene cyanol, and denatured at 95°C for 5 min. SSCP analysis was performed by electrophoresing denatured samples through a non-denaturing 6% polyacrylamide gel at 10 W constant power at either 4°C or 24°C in the presence of 5% glycerol (Cama *et al.*, 1993).

DNA samples showing an abnormal SSCP electrophoretic profile were further analysed by direct sequencing of PCR products using the dideoxy chain-termination method (Kadowaki *et al.*, 1990).

### Results and discussion

SSCP analysis of *APC* sequences showed an altered band pattern only in one case of multifocal papillary carcinoma (Figure 1, lane 7). This alteration was observed in the exon 15 region corresponding to codons 1,389-1,547 and was also present in a different DNA sample obtained from a distinct focus of the same neoplastic lesion (Figure 1, lane 27). No alterations in the *APC* sequence were detected in the thyroid extratumoral tissue obtained from the same patient (Figure 1, lane 28) or in non-tumoral specimens from any of the other patients (data not shown). Direct sequencing of the two samples demonstrated, in both cases, the presence of a guanine to adenine transition at nucleotide position 4,497 of the *APC* coding sequence, corresponding to a CG dinucleotide. This nucleotide change, however, represented a silent mutation since it did not cause any amino acid change in the primary structure of the APC protein (data not shown). The

**Figure 1** SSCP analysis of *APC* gene exon 15. A portion of exon 15 of the *APC* gene (codons 1,389–1,547) was amplified by PCR and scanned by SSCP for the presence of mutations in 13 papillary (lanes 1–13), three follicular (lanes 14–16) and two anaplastic (lanes 17 and 18) carcinomas and in eight follicular adenomas (lanes 19–26). In lane 27 is shown the SSCP profile of DNA extracted from a different focus of the same papillary tumour shown in lane 7. The altered SSCP pattern (lanes 7 and 27) reflected a conservative nucleotide substitution that was not present in the extratumoral tissue from the same thyroid gland (lane 28) or in two other non-neoplastic samples (lanes 29–30).

remaining samples did not show alterations of the *APC* gene in any of the mutation cluster regions analysed (Figure 1).

Since 1949 the concurrence of FAP and thyroid carcinoma has been observed in 48 cases (Table I). Most of the studies published have pointed to the fact that thyroid carcinoma occurs with an unexpectedly high frequency in patients affected by FAP. Recently, statistical analysis of data obtained from an English Polyposis Register has indicated that the risk for a young female affected by FAP of developing thyroid carcinoma, particularly of the papillary type, is about 160-fold higher than expected (Plail *et al.*, 1987). Similar conclusions have been obtained in a Danish population of FAP patients in which the risk of developing thyroid carcinoma has also been estimated to be 100-fold greater than in the general population (Bülow *et al.*, 1988). Thyroid carcinoma associated with FAP has been more frequently found in young female patients (F/M = 6.3:1) than sporadic thyroid carcinoma (F/M = 2–3:1). The thyroid neoplasia has usually been discovered within 1–7 years after FAP was diagnosed. Papillary carcinoma represented the predominant histotype (8.5%) with a 2-fold higher than expected frequency of multifocal lesions (Table I).

The patients with thyroid carcinoma examined in the present study were all females, did not show evidence of an altered bowel function and always had a negative family

**Table I** Association between familial adenomatous polyposis (FAP) and thyroid carcinoma (TC)

| Case | Reference | Sex | Age TC | Age FAP | Histological type of thyroid carcinoma |
|---|---|---|---|---|---|
| 1 | Crail (1949) | M | 24 | 24 | Papillary |
| 2 | Ogata *et al.* (1964) | M | 31 | – | Adenocarcinoma |
| 3 | Raynham & Louw (1966) | F | 20 | 20 | Unknown (multifocal) |
| 4 | Smith (1968) | M | 29 | 39 | Papillary (multifocal) |
| 5 | | – | – | – | Unknown |
| 6 | | – | – | – | Unknown |
| 7 | Camiel *et al.* (1968) | F | 19 | 28 | Papillary |
| 8 | | F | 20 | 29 | Papillary (multifocal) |
| 9 | Smith & Kern (1973) | F | 19 | 26 | Papillary (multifocal) |
| 10 | Alm & Licznersci (1973) | F | – | – | Unknown |
| 11 | | F | – | – | Unknown |
| 12 | | – | – | – | Unknown |
| 13 | | – | – | – | Unknown |
| 14 | Mathias & Smith (1977) | F | <30 | – | Papillary |
| 15 | Keshgegian & Enterline (1978) | F | 21 | 14 | Papillary (multifocal) |
| 16 | Takahashi *et al.* (1976) | M | 58 | – | Papillary |
| 17 | Iida *et al.* (1977) | M | 26 | – | Unknown |
| 18 | | F | 26 | – | Unknown |
| 19 | Ushio *et al.* (1977) | M | 27 | – | Unknown |
| 20 | Harada *et al.* (1977) | F | 22 | – | Papillary |
| 21 | Okamura *et al.* (1979) | F | 29 | – | Papillary |
| 22 | Hamilton *et al.* (1979) | F | 18 | 17 | Papillary |
| 23 | Miura *et al.* (1980) | F | 27 | – | Papillary |
| 24 | Lee & Mackinnon (1981) | F | 23 | 32 | Papillary (multifocal) |
| 25 | Delamarre *et al.* (1982) | F | 21 | 27 | Follicular |
| 26 | Thompson *et al.* (1983) | F | 24 | 22 | Papillary (multifocal) |
| 27 | Schneider *et al.* (1983) | F | 37 | 33 | Papillary |
| 28 | Masuyama *et al.* (1986) | F | 26 | – | Papillary |
| 29 | Plail *et al.* (1987) | F | 22 | 21 | Papillary (multifocal) |
| 30 | | F | 26 | 19 | Papillary (multifocal) |
| 31 | | F | 34 | 31 | Papillary |
| 32 | | F | 23 | 27 | Papillary (multifocal) |
| 33 | | F | 20 | 20 | Unknown (multifocal) |
| 34 | | F | 16 | 28 | Unknown |
| 35 | | F | 34 | 17 | Unknown |
| 36 | Piffer (1988) | F | 26 | 24 | Follicular (multifocal) |
| 37 | | F | 28 | – | Papillary |
| 38 | | F | 55 | – | Unknown |
| 39 | Delamarre *et al.* (1988) | F | 29 | 16 | Papillary (multifocal) |
| 40 | | F | 26 | 21 | Papillary (multifocal) |
| 41 | Bülow *et al.* (1988) | F | 19 | 17 | Papillary |
| 42 | | F | – | – | Papillary |
| 43 | | F | 40 | 26 | Papillary |
| 44 | van Erpecum *et al.* (1988) | F | 34 | 31 | Papillary |
| 45 | Herrera *et al.* (1989) | F | 27 | 23 | Follicular |
| 46 | | F | – | – | Papillary |
| 47 | Ono *et al.* (1991) | F | 50 | 50 | Follicular |
| 48 | Bell & Mazzaferri (1993) | F | 24 | 24 | Papillary |

history for the presence of gastrointestinal diseases. Only one among the 13 cases of papillary thyroid carcinoma was multifocal. In this case, the occurrence of the same mutation in DNA extracted from two distinct tumoral foci, localized in opposite lobes of the same thyroid gland, but not in the corresponding extratumoral tissue (Figure 1, lane 28), suggested a common clonal origin of the thyroid tumour.

The frequent association between FAP and thyroid carcinoma suggests the involvement of common mechanisms in the pathogenesis of these two diseases. However, the absence of germline and somatic *APC* gene defects in our series of 26 thyroid neoplasms, at different stages of dedifferentiation, suggests that alterations of this tumour-suppressor gene do not represent a frequent event in thyroid tumorigenesis.

The small number of cases analysed does not allow any statistically significant conclusion to be drawn, since a low incidence of *APC* mutations cannot be ruled out. However, our results are in agreement with recent unpublished observations (Varesco *et al.*, 1993; Zeki *et al.*, 1993). SSCP analysis of a population of 73 benign and malignant thyroid tumours, performed on a 1,200 bp stretch of exon 15, also failed to detect any mutation in the *APC* gene. Only a nucleotide insertion, leading to a premature stop codon, was identified in one out of four thyroid carcinoma cell lines examined, namely the highly undifferentiated ARO carcinoma cells (Zeki *et al.*, 1993). *APC* gene mutations were also absent in a small group of thyroid tumours in which most of the *APC* gene coding region was investigated (Varesco *et al.*, 1993).

In conclusion, our own results and those of others, gathered from a total of more than 100 thyroid tumours, suggest that *APC* mutations do not play a pathogenetic role in thyroid tumorigenesis, at least in patients not affected by FAP. However, true estimates of the incidence of *APC* alterations in thyroid tumours will require the collection of molecular information from a larger number of cases.

This work has been supported by research grants from Associazione Italiana per la Ricerca sul Cancro (AIRC), from Ministero dell' Università e della Ricerca Scientifica e Tecnologica (MURST) and from Consiglio Nazionale della Ricerche (CNR).

### References

ALM, T. & LICZNERSCI, G. (1973). The intestinal polyposes. *Clin. Gastroenterol.*, **2**, 577–602.

BELL, B. & MAZZAFERRI, E. (1993). Familial adenomatous polyposis (Gardner's syndrome) and thyroid carcinoma. *Dig. Dis. Sci.*, **38**, 185–190.

BÜLOW, S., HOLM, N.V. & MELLEMEMGAARD, A. (1988). Papillary thyroid carcinoma in Danish patients with familial adenomatous polyposis. *Int. J. Colorect. Dis.*, **3**, 29–31.

CAMA, A., PALMIROTTA, R., ESPOSITO, D., CURIA, M.C., RANIERI, A., FICARI, F., VALANZANO, R., BATTISTA, P., FRATI, L., TONELLI, F. & MARIANI CONSTANTINI, R. (1993). A novel deletion in exon 15 of the adenomatous polyposis coli gene in an Italian kindred. *Hum. Mutat.* (in press).

CAMIEL, M.R., MULE', J.E., ALEXANDER, L.L. & BENNINGHOFF, D.L. (1968). Association of thyroid carcinoma with Gardner'syndrome in siblings. *N. Engl. J. Med.*, **278**, 1056–1058.

CRAIL, H.W. (1949). Multiple primary malignancy arising in the rectum, brain and thyroid. Report of a case. *US Navy Med. Bull.*, **49**, 123–128.

DELAMARRE, J., DUPAS, J.L., CAPRON, J.P., ARMAND, A., HERVE, M. & DESCOMBES, P. (1982). Polypose rectocolique familiale, syndrome de Gardner et cancer thyroidien: estude de deux cas. *Gastroenterol. Clin. Biol.*, **6**, 1016–1019.

DELAMARRE, J., CAPRON, J.P., ARMAND, A., DUPUS, J.L., DESCHEPPER, B. & DAVION, T. (1988). Thyroid carcinoma in two sisters with familial polyposis of the colon: case report and review of the literature. *J. Clin. Gastroenterol.*, **10**, 659–662.

DONGHI, R., LONGONI, A., PILOTTI, S., MICHIELI, P., DELLA PORTA, G. & PIEROTTI, M.A. (1993). Gene p53 mutations are restricted to poorly differentiated and undifferentiated carcinomas of the thyroid gland. *J. Clin. Invest.*, **91**, 1753–1760.

FAGIN, J.A., MATSUO, K., KARMAKAR, A., CHEN, D.L., TANG, S.-H. & KOEFFLER, H.P. (1993). High prevalence of mutations of the p53 gene in poorly differentiated human thyroid carcinomas. *J. Clin. Invest.*, **91**, 179–184.

GARDNER, E.J. & RICHARDS, R.C. (1953). Multiple cutaneous and subcutaneous lesions occurring simultaneously with hereditary polyposis and osteomatosis. *Am. J. Genet.*, **5**, 139–147.

HAMILTON, R.S., BUSSEY, H.J.R., MENDELSHON, G., DIAMOND, M.P., PAVLIDES, G., HUTCHEON, D., HARBINSON, M., SHERMETA, D., MORSON, B.C. & YARDLEY, J.H. (1979). Ileal adenomas after colectomy in nine patients with adenomatous polyposis coli/Gardner's syndrome. *Gastroenterology*, **77**, 1252–1257.

HARADA, M., MURAKAMI, T., SHISHIDO, Y., HARADA, R., ITOH, S., KONN, M., HORIMAI, T. & FUJITA, H. (1977). Two cases of unusual extracolonic phenotypes accompanying familial polyposis of colon – one with papillary carcinoma of the thyroid and other with mesenteric fibromatosis. *Nippon Shokaki Byou Gakkai Zasshi (Jpn. J. Gastroenterol.)*, **74**, 1567–1574.

HERRERA, L., CARREL, A., RAO, U., CASTILLO, N. & PETRELLI, N. (1989). Familial adenomatous polyposis in association with thyroiditis: report of two cases. *Dis. Colon Rectum*, **10**, 893–896.

HORII, A., NAKATSURU, S., ICHII, S., NAGASE, H. & NAKAMURA, Y. (1993). Multiple forms of the APC-gene transcripts and their tissue-specific expression. *Hum. Mol. Genet.*, **2**, 283–287.

IIDA, M., YAO, Y., OHGUSHI, H, OMAE, T., OHSATO, K., ITOH, H., WATANABE, H. & MIZUTANI, T. (1977). Gastric lesions in familial adenomatosis of the colon; on their characteristics and follow up studies. *I To Cho (Stom. Intest.)*, **12**, 1365–1374.

ITO, T., SEYAMA, T., MIZUNO, T., TSUYAMA, N., HAYASHI, T., HAYASHI, Y., DOHI, K., NAKAMURA, N. & AKIYAMA, M. (1992). Unique association of p53 mutations with undifferentiated but not with differentiated carcinomas of the thyroid gland. *Cancer Res.*, **52**, 1369–1371.

JAGELMAN, D.G., DECOSSE, J.J., BUSSEY, H.J.R. & THE LEEDS CASTLE POLYPOSIS GROUP (1988). Upper gastrointestinal cancer in familial adenomatous polyposis. *Lancet*, **i**, 1149–1151.

JÄRVINEN, H.J. & SIPPONEN, P. (1986). Gastroduodenal polyps in familial adenomatous and juvenile polyposis. *Endoscopy*, **18**, 230–234.

KADOWAKI, T., KADOWAKI, H. & TAYLOR, S.I. (1990). A nonsence mutation causing decreased levels of insulin receptor mRNA: detection by a simplified technique for direct sequencing of genomic DNA amplified by polymerase chain reaction. *Proc. Natl Acad. Sci. USA*, **87**, 658–662.

KESHGEGIAN, A.A. & ENTERLINE, H.T. (1978). Gardner's syndrome with duodenal adenomas, gastric adenomyoma and thyroid papillary-follicular adenocarcinoma. *Dis. Colon Rectum*, **21**, 255–260.

LEE, F.I. & MACKINNON, M.D. (1981). Papillary thyroid carcinoma associated with polyposis coli. *Am. J. Gastroenterol.*, **76**, 138–140.

MASUYAMA, K., KAWAHARA, M., MORI, T., HAYASHI, K., TOMINAGA, K. & TANAKA, R. (1986). An atypical case of familial adenomatous polyposis with multiple thyroid cancers and colonic cancers. *Nippon Shokaki Geka Gakkai Zasshi (Jpn. J. Gastroenterol. Surg.)*, **19**, 1316.

MATHIAS, J.R. & SMITH, W.G. (1977). Mesenteric fibromatosis associated with familial polyposis. *Am. J. Dig. Dis.*, **22**, 741–744.

MIURA, K., YAMAGUCHI, A., KAWASE, K., KONDO, S., IWASE, K., FUKUKEI, I. & IEDA, H. (1980). A case of adenomatous coli associated with carcinoma of the colon, ovary and thyroid gland and nodular hyperplasia of the adrenal cortex. *Nippon Shokaki Geka Gakkai Zasshi (Jpn. J. Gastroenterol. Surg.)*, **13**, 328–332.

MIYOSHI, Y., NAGASE, M., ANDO, M., HORH, A., ICHO, S., NAKATSURU, S., AOKI, T., MIKI, Y., MORI, T. & NAKAMURA, Y. (1992). Somatic mutations of the APC gene in colorectal tumors: mutation cluster region in the APC gene. *Hum. Mol. Genet.*, **1**, 229–233.

NAKAMURA, T., YANA, I., KOBAYASHI, T., SHIN, E., KARAKAWA, K., FUJITA, S., MIYA, A., MORI, T., NISHISHO, I. & TAKAI, S.I. (1992). p53 gene mutations associated with anaplastic transformation of human thyroid carcinomas. *Cancer Res.*, **83**, 1293–1298.

NAKATSURU, S., YANAGISAWA, A., ICHII, S., TAHARA, E., KATO, Y., NAKAMURA, Y. & HORII, A. (1992). Somatic mutation of the APC gene in gastric cancer; frequent mutations in very well differentiated adenocarcinoma and signet-ring cell carcinoma. *Hum. Mol. Genet.*, **1**, 559–563.

OGATA, H., OHTSUKA, K. & TANAKA, K. (1964). A case of rectal cancer which presented an interesting metastasis. *Bouei Eiseii (Natl. Def. Med. J.)*, **11**, 137–138.

OKAMURA, S., KUSUGAMI, K., KUROKAWA, S., MIWA, M., OKA, Y. & HATTORI, T. (1979). A case of familial polyposis coli with interesting findings. *Saishin Igaku*, **34**, 2697–2700.

ONO, C., IWAMA, T. & MISHIMA, Y. (1991). A case of familial adenomatous polyposis complicated by thyroid carcinoma of the ampulla of Vater and adrenocortical adenoma. *Jpn J. Surg.*, **21**, 234–240.

PIFFER, S. (1988). Gardner's syndrome and thyroid cancer a case report and review of the literature. *Acta Oncol.*, **27**, 413–415.

PLAIL, R.O., BUSSEY, H.J.R., GLAZER, R. & THOMSON, J.P.S. (1987). Adenomatous polyposis: an association with carcinoma of the thyroid. *Br. J. Surg.*, **74**, 377–380.

POWELL, S.M., NIEY, N., BEAZER BARCLAY, Y., BRYAN, T.M., HAMILTON, S.R., THIBODEAN, S.N., VOGELSTEIN, B. & KINGLER, K.W. (1992). APC mutation occur early during colorectal tumorigenesis. *Nature*, **359**, 235–237.

RAYNHAM, W.H. & LOUW, J.H. (1966). Familial polyposis of the colon. *S. Afr. Med.*, **40**, 857–865.

SCHNEIDER, N.R., CUBILLA, A.L. & CHAGANTI, R.S.K. (1983). Association of endocrine neoplasia with multiple polyposis of the colon. *Cancer*, **51**, 1171–1175.

SMITH, W.G. (1968). Familial multiple polyposis: research tool for investigating the etiology of carcinoma of the colon. *Dis. Colon Retcum*, **11**, 17–31.

SMITH, W.G. & KERN, B.B. (1973). The nature of the mutation in familial multiple polyposis: papillary carcinoma of the thyroid, brain tumors and familial multiple polyposis. *Dis. Colon Rectum*, **16**, 264–271.

TAKAHASHI, S., OKUNO, Y., NAKAMURA, A., JINN, S., ONO, Y., SHINOMURA, T., KUWATA, Y. & SETA, K. (1976). Gardner's syndrome in association with thyroid carcinoma. *Rinsho Geka (J. Clin. Surg.)*, **31**, 795–800.

THOMPSON, J.S., HARNED, R.K., ANDERSON, J.C. & HODGSON, P.E. (1983). Papillary carcinoma of the thyroid and familial polyposis coli. *Dis. Colon Rectum*, **26**, 583–585.

USHIO, K., ABE, S., MITSUSHIMA, T., KIMURA, T., MORIYAMA, N., TAKASUGI, T., OKAZAKI, M., MATSUE, N., SASAGAWA, M., YAMADA, T., OGURO, Y., KODAIRA, S., HOJO, K., KOYAMA, Y., ITABASHI, M., HIROTA, E. & ISHIKAWA, H. (1977). Gastric and duodenal lesions associated with familial polyposis coli. *I To Cho (Stom. Intest.)*, **12**, 1547–1557.

VAN ERPECUM, K.J., VAN BERGE HENEGOUWEN, G.P., MEINDERS, A.E. & BRONKHORST, F.B. (1988). Papillary thyroid carcinoma and characteristic pigmented ocular fundus lesions in a patient with Gardner's syndrome. *Neth. J. Med.*, **32**, 136–142.

VARESCO, L., GISMONDI, I., DE BENEDETTI, L., BAFICO, A. & FERRARA, G.B. (1993). Germline and somatic mutations of the adenomatous polyposis coli (APC) gene. First Italian Congress on Molecular Oncology, Positano, Italy. *Abstract Book*, p. 121.

ZEKI, K., TANG, S.-H., GONSKI, R. & FAGIN, J.A. (1993). Mutations of the adenomatous polyposis coli gene in sporadic thyroid neoplasms. 65th Meeting of the American Thyroid Association, Tampa, Florida. *Thyroid*, **3** (Suppl.), T111.